# Supplementary material for: Influence of Storage Temperature on Starch Retrogradation and Digestion of Chinese Steamed Bread
Source: Foods. 2024 Feb 7;13(4):517. doi: 10.3390/foods13040517 (PMC10888248; doi:10.3390/foods13040517)
Supplement: Supplementary file 1 [file foods-13-00517-s001.zip › foods-2811685-supplementary.pdf]

Influence of storage temperature on starch retrogradation and digestion of Chinese steamed bread

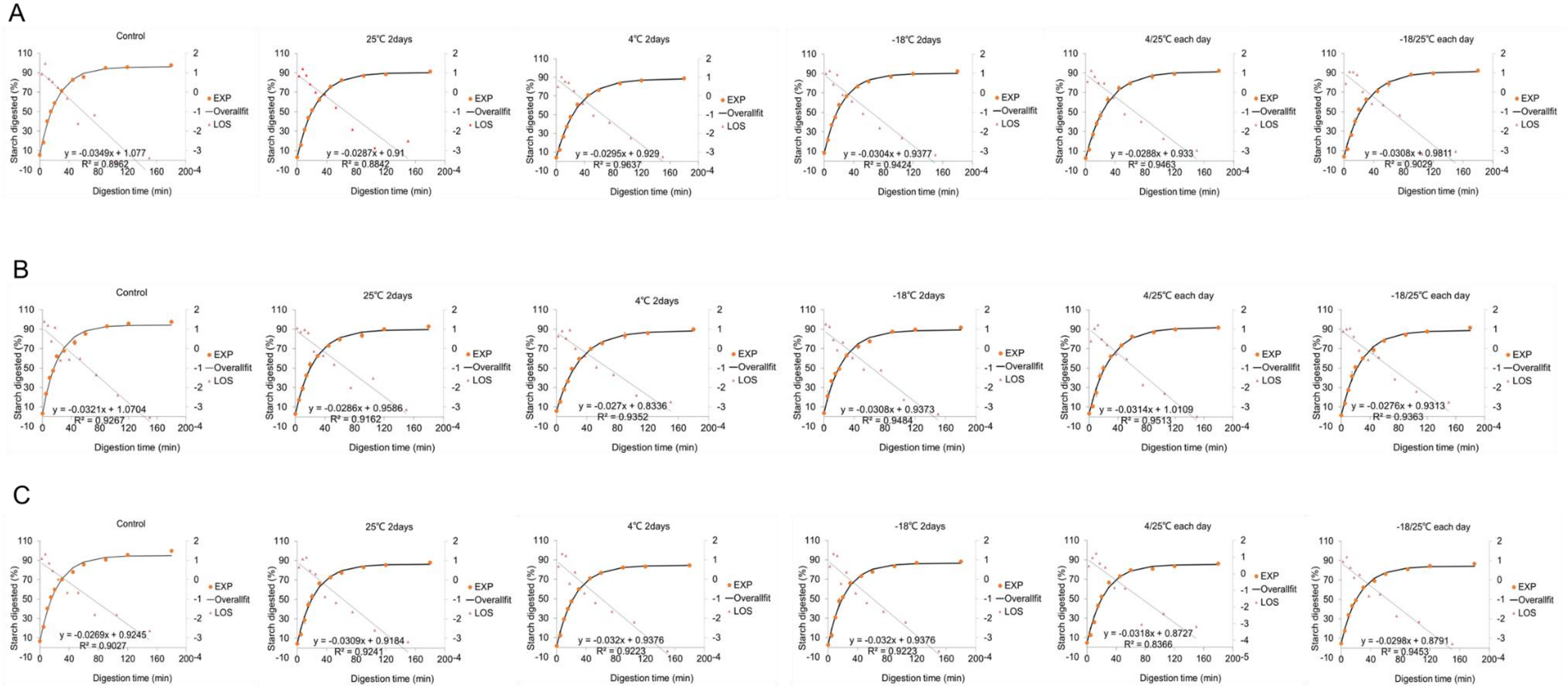

Figure S1. LOS plots and first-order kinetics fittings for starch digestograms of CSB stored at different temperatures (A: Yongliang4; B: Xiaoyan6; C: Xiaoyan22).
